# Selenium-transition metal supported on a mixture of reduced graphene oxide and silica template for water splitting†

**DOI:** 10.1039/d3ra01945d

**Published:** 2023-05-25

**Authors:** R. S. Amin, Amani E. Fetohi, D. Z. Khater, Jin Lin, Yanzhong Wang, Chao Wang, K. M. El-Khatib

**Affiliations:** a Chemical Engineering Department, Engineering Research and Renewable Energy Institute, National Research Centre 33 El-Buhouth St., Dokki Cairo 12622 Egypt kamelced@hotmail.com; b School of Materials Science and Engineering, North University of China Taiyuan 030051 China

## Abstract

Exploration of economical, highly efficient, and environment friendly non-noble-metal-based electrocatalysts is necessary for hydrogen and oxygen evolution reactions (HER and OER) but challenging for cost-effective water splitting. Herein, metal selenium nanoparticles (M = Ni, Co & Fe) are anchored on the surface of reduced graphene oxide and a silica template (rGO-ST) through a simple one-pot solvothermal method. The resulting electrocatalyst composite can enhance mass/charge transfer and promote interaction between water molecules and electrocatalyst reactive sites. NiSe_2_/rGO-ST shows a remarkable overpotential (52.5 mV) at 10 mA cm^−2^ for the HER compared to the benchmark Pt/C E-TEK (29 mV), while the overpotential values of CoSeO_3_/rGO-ST and FeSe_2_/rGO-ST are 246 and 347 mV, respectively. The FeSe_2_/rGO-ST/NF shows a low overpotential (297 mV) at 50 mA cm^−2^ for the OER compared to RuO_2_/NF (325 mV), while the overpotentials of CoSeO_3_-rGO-ST/NF and NiSe_2_-rGO-ST/NF are 400 and 475 mV, respectively. Furthermore, all catalysts indicate negligible deterioration, indicating better stability during the process of HER and OER after a stability test of 60 h. The water splitting system composed of NiSe_2_-rGO-ST/NF||FeSe_2_-rGO-ST/NF electrodes requires only ∼1.75 V at 10 mA cm^−2^. Its performance is nearly close to that of a noble metal-based Pt/C/NF||RuO_2_/NF water splitting system.

## Abstract 2

Exploration of economical, highly efficient, and environment friendly non-noble-metal-based electrocatalysts is necessary for hydrogen and oxygen evolution reactions (HER and OER) but challenging for cost-effective water splitting.

## Graphical abstract

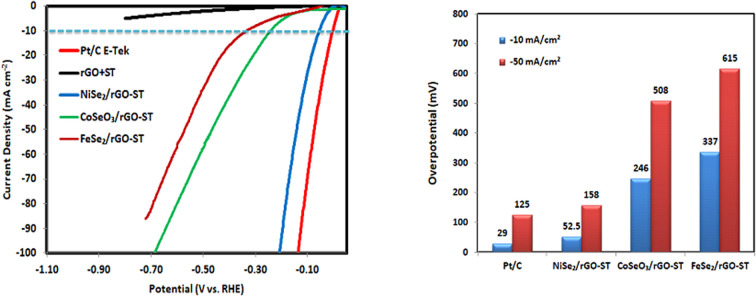

## 1. Introduction

Water electrolysis is considered a zero-carbon approach for producing hydrogen; hence there is no consumption of fossil fuels or CO_2_ emissions.^1^ Hydrogen is a clean and efficient energy source that may be used to generate electricity in the stationary industrial and domestic and automotive sectors.^2–4^ There are two main approaches for low temperature water electrolysis: liquid alkaline using a KOH electrolyte and polymer electrolyte membrane using a solid ion exchange membrane, depending on the kind of electrolyte. The most applicable kind of both is the alkaline one. This technology is designed for long-term stable operation.^5^ Water electrolysis is an electrochemical process that involves two half–cell reactions: hydrogen evolution reaction (HER) and oxygen evolution reaction (OER). Because of the high energy barrier for H^+^ reduction and O_2_ oxidation in a water molecule, the efficiency of both processes is very low. As a direct result, the development of various kinds of electrocatalysts to facilitate both reactions is a very important issue.^6–9^ Nowadays, the most efficient electrocatalysts for water electrolysis are the noble metals Ir or Ru^10^ and their oxides for OER,^11^ and Pt for HER.^6^ However, noble metals are very rare and expensive.^12^ Therefore, exploring low cost and efficient electrocatalysts is vital for water electrolysis technology.^13,14^ Transition metal-based electrocatalysts, such as oxides,^15^ hydroxides,^16^ carbides,^17^ phosphides,^18^ sulfides,^19^ and chalcogenides,^20^ are considered promising electrocatalysts to replace noble-metal ones. Transition-metal selenides have important applications in electrochemical systems and have attracted extensive attention in water electrolysis, owing to their chemical stability, high conductivity, and efficient catalytic activity.^21,22^

It is well known that nickel and iron diselenide electrocatalysts have good performance in water-splitting reactions.^23,24^ Lingling Zhai *et al.*^25^ have prepared NiSe_2_ supported on carbon fiber paper by pyrolysis and selenization reactions, the electrocatalyst exhibited overpotential values of 145 and 280 mV at a current density of 10 mA cm^−2^ for HER and OER respectively in an alkaline medium,. Moreover, iron-doping NiSe_2_ supported on Ni–Fe foam is synthesized by Changqin Zhang *et al.*^26^ by oxalic immersion and selenization method. The overpotential is 145 mV for HER and 135 mV for OER. Jing Yu *et al.*^27^ prepared a P-NiSe_2_@N-CNTs/NC hybrid catalyst that is prepared by phosphorization and selenization, with an overpotential of 95 and 306 mV gained at 10 mA cm^−2^ for HER and OER respectively in alkaline media. Furthermore, the hydrothermal method used to prepare FeSe_2_ nanoparticles embedded in a selenium matrix by Fanjun Kong *et al.*^28^. They tested its electrochemical performance for OER in an alkaline medium and they found that Tafel slopes of FeSe_2_ and FeSe_2_@Se-2 are 70 and 54 mV dec^−1^ respectively. Tafel slope is a measure of the overall kinetics of HER and OER so a lower slope means a faster reaction. Huixuan Zhang *et al.*^24^ prepared FeSe_2_/CoFe_2_O_4_ by two steps hydrothermal method, it gained a Tafel slope of 88.76 mV dec^−1^ and that of FeSe_2_ is 107.9 mV dec^−1^ for HER in an acidic medium. In addition, X. Xing *et al.*^29^ found an easy way to synthesize bimetallic transition metal selenides electrocatalysts for water-splitting reactions, they prepared FeSe_2_/CoSe nanosheet by hydrothermal and selenization processes and tested it in an alkaline medium for water electrolysis. The results reviled that, at 10 mA cm^−2^, low overpotential of 73 mV for HER and 183 mV for OER. Moreover, Beibei Sun *et al.*^30^ prepared MnS_*x*_Se_1−x_@N,F-CQDs using microwave-assisted heating, hydrothermal and calcination and tested it as bifunctional electrocatalyst for water electrolysis in an alkaline medium. They found that the overpotential at 10 mA cm^−2^ of 209 mV for OER and 87 mV for HER. Xiaowei Pan *et al.*^31^ developed a CoSe_2_ nanorods and selenium vacancies using a plasma cleaner (CoSe_2_–VSe/CC) to enhance their catalytic activity. The catalyst exhibited a lower overpotential of 88 mV at 10 mA cm^−2^ in 1 M KOH and high durability over 100 h at 100 mA cm^−2^.

In another point of view, CoSeO_3_ which is a transition metal selenite, has gained great interest in lithium-ion batteries.^32^ Moreover, CoSe_2_/CoSeO_3_ is tested as an electrocatalyst in dye-sensitized solar cells, it is obtained by a micro emulsion-assisted hydrothermal synthesis.^33^ ZHAO Dong-Jiang *et al.*^34^ prepared CoSeO_3_ nanoparticles by low-temperature refluxing method, they found that the electrocatalyst has good electrocatalytic activity towards oxygen reduction reaction. Yu Zhou *et al.*^35^ synthesized a Ni–Co_4_S_3_ (Sv)/N-V_2_CT_*x*_ electrocatalysts by sulfuration and ultrasonic treatment, the performance shows that the electrocatalyst has an overpotential of 127 mV at 10 mA cm^−2^ for HER.

To further enhance the electrochemical efficiencies of the prepared electrocatalysts towards water splitting, reduced graphene oxide (rGO), is utilized as a support material. rGO has a lot of good characteristics as low electrical resistance, high stability, and large surface area.^36^ Another potential support material is hollow silica spheres; it has a mesoporous structure which enables it to be used in catalysis and drug storage applications. In this paper, reduced graphene oxide and silica templates were prepared and used as supporting materials for the electrocatalysts, NiSe_2_, FeSe_2,_ and CoSeO_3_ synthesized by hydrothermal technique. Integration between the supporting materials with transition metal-diselined (NiSe_2_ and FeSe_2_) and a transition metal selenite (CoSeO_3_) has occurred. The prepared electrocatalysts have been tested for water-splitting reactions in an alkaline medium.

In this study, we report a one-pot solvo thermal method synthesis strategy to prepare a bifunctional electrocatalyst composed of Se and transition metals (*i.e.* Co, Ni, and Fe) supported on a mixture of rGO & ST. The atomic ratio of selenium to the other transition metals is calculated to be 1 : 1 where the total loading of the electrocatalysts on the support is fixed at 30%. The benefits of integrated structural design in delivering remarkable bi-functional activities for HER/OER. A water-splitting system composed of NiSe_2_-rGO-ST/NF||FeSe_2_-rGO-ST/NF electrodes requires only ∼1.75 V at 10 mA cm^−2^. Its performance is comparable to that of a noble metal-based Pt/C/NF||RuO_2_/NF water splitting system. Our research demonstrates a simple method for creating bifunctional electrocatalysts for hydrogen production.

## 2. Materials & methods

### 2.1. Materials

All chemicals are obtained in analytical grade from Sigma-Aldrich (USA) and are used without any purification. All aqueous solutions, including preparation and cleaning, are made using double-distilled water.

### 2.2. Support preparation

#### 2.2.1. Graphene oxide preparation

**Scheme 1:**
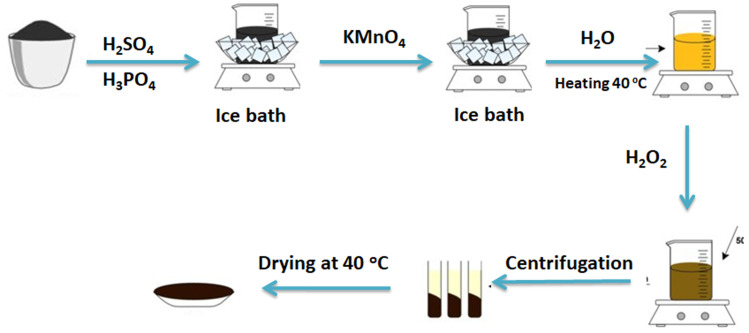
Procedure of graphene oxide preparation.

Graphene oxide (GO) is prepared following a modified Hummers' method as shown in Scheme 1.^37^ 5 g of graphite powder is put in a round flask (capacity is 2000 mL) then 360 mL of concentrated H_2_SO_4_ and 40 mL of phosphoric acid are added to the graphite under stirring at room temperature for one hour. Afterward, the round flask is placed in an ice bath. After cooling, the mixture of 18 g of potassium permanganate is added slowly under continuous stirring (1 g each 10 min). After finishing the addition of potassium permanganate powder, the mixture remains in the ice bath for 10 minutes. Then the produced mixture is warmed up to 40 °C and maintained at this temperature for two and half hours in a water bath resulting in a thick paste. Subsequently, an additional 500 mL of water is followed by the addition of 30 mL of hydrogen peroxide (30%). Afterward, 250 mL of 10% hydrochloric acid is added to the mixture. Lastly, the mixture is rinsed and centrifuged many times to eliminate supernatant contaminants until the pH of the supernatant water reaches 5.5. Then the mixture is dried in a hot air oven at 80 °C for six hours.

#### 2.2.2. Silica template preparation

The silica template is synthesized by mixing ammonia-catalyzed hydrolysis of tetraethoxysilane (TEOS) in ethanol–water solvents.^38^ The procedure of preparation is as follows: 8 mL of double-distilled water and 8 mL of ammonia are added into 200 mL of ethanol mixed with 10 mL of TEOS solution. The mixture is stirred continuously for 24 hours then it is filtered and dried in a dryer at 80 °C for 6 hours.

#### 2.2.3. Mixed GO and silica template support preparation

An appropriate amount of prepared graphene oxide is added to a 200 mL aqueous solution of ST under intense magnetic stirring for 1 h. The product is obtained by filtration and washing with double distilled water. The resulting mixture dried in a dryer at 80 °C for 6 h.

### 2.3. Preparation of selenium-transition metal supported on a mixture of rGO and ST

**Scheme 2:**
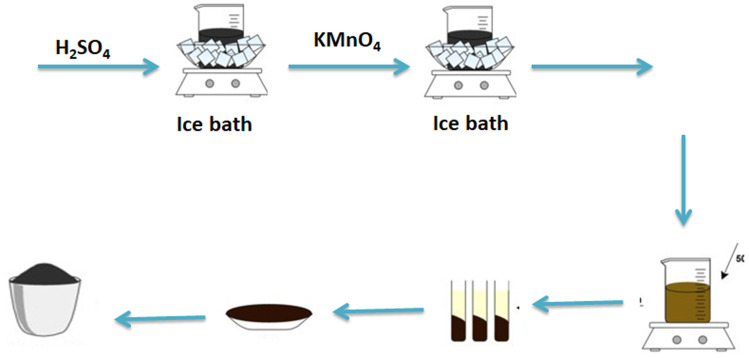
Procedure of selenium-transition metal supported on rGO and ST preparation.

The electrocatalysts are prepared using the one-pot solvo thermal method as shown in Scheme 2. The bimetallic electrocatalysts are composed of Se and transition metals (*i.e.* Co, Ni, and Fe) supported on a mixture of rGO & ST. The atomic ratio of selenium to the other transition metals is calculated to be 1 : 1 where the total loading of the electrocatalysts on the support is fixed at 30%. Chloride salts are used as the precursors for the transition metals. For a typical synthesis, appropriate amounts of Se and NaBH_4_ (it acted as a reducing agent) are dissolved into 50 mL *N*,*N*-dimethyl formamide (DMF). The mixture is then agitated until a uniformly distributed black-color solution is formed. Afterward, calculated amounts of metal chlorides are added to this black-color solution. The produced solution is then placed in a Teflon-lined autoclave and heated at 160 °C for 24 hours. After cooling naturally, the acquired products are filtrated and washed with double distilled water several times and dried in a dryer at 60 °C for 6 hours. According to the procedure mentioned above, the electrocatalysts prepared are namely as follows: CoSeO_3_/rGO-ST, NiSe_2_/rGO-ST, and FeSe_2_/rGO-ST.

### 2.4. Physical characterization of the electrocatalysts

The crystalline phase is analyzed *via* a Rigaku-D/MAX-PC 2500 X-ray diffraction (XRD) system. Scanning electron microscopy (SEM) and high-resolution transmission electron microscopy (HRTEM, JEOL-JEM 2010) are utilized to investigate microstructure and morphology. X-ray photoelectron spectroscopy (XPS) (PH1-5700 ESCA system, US) is used to determine the valence states of the electrocatalyst.

### 2.5. Electrochemical characterization

#### 2.5.1. Electrode preparation

The catalyst and polyvinylidene fluoride (PVDF) are blended in *N*-methyl-2-pyrrolidone (NMP) solvent with a ratio of 9 : 1 by weight to obtain catalyst slurry. Then, the mixture is treated by sonication for more than 1 h until the slurry becomes a uniform dispersion nickel foam (1 cm × 2 cm) is cleaned by sonication in HCl (3 M) for 15 min, deionized water, and ethanol, respectively, and then dried in a vacuum oven at 60 °C for 8 h. The slurry is drop cast on glassy carbon 3 mm diameter (0.07 cm^2^) for hydrogen evolution reaction, and 1 × 1 cm^2^ nickel foam to make a working electrode for oxygen evolution reaction (the loading mass is about 1 mg cm^−2^).

#### 2.5.2. Electrochemical characterization of electrocatalyst

The electrocatalytic activities are evaluated using a three-electrode setup equipped with an electrochemical workstation Voltalab6. The counter electrode and reference electrodes are platinum wire and a KCl-saturated Ag/AgCl electrode, respectively. Polarization curves for HER and OER are measured using linear sweep voltammetry (LSV) at a scan rate of 5 mV s^−1^ in 1.0 M KOH and corrected with *iR* compensation. Electrochemical impedance spectroscopy (EIS) measurement is performed in a frequency range of 0.1–10^5^ Hz with a polarized potential in the turnover region of the HER and OER. All potentials are corrected to the reversible hydrogen electrode (RHE) according to the Nernst equation: *E*_RHE_ = *E*(Ag/AgCl) + 0.197 + 0.059 × pH. The electrochemically active surface areas (ECSA) of as-obtained electrocatalysts are achieved from the corresponding electrochemical double-layer capacitance (*C*_dl_), which can be calculated from CV curves. Cyclic voltammograms (CV) are recorded with increasing scan rates (20–140 mV s^−1^) within the non-faradaic potential region (0.67–0.77 V *vs* RHE) for HER & (1.2–1.3 V *vs.* RHE) for OER.

## 3. Results & discussion

### 3.1. Physical characterization

#### 3.1.1. Physical characterization of support

**Fig. 1:**
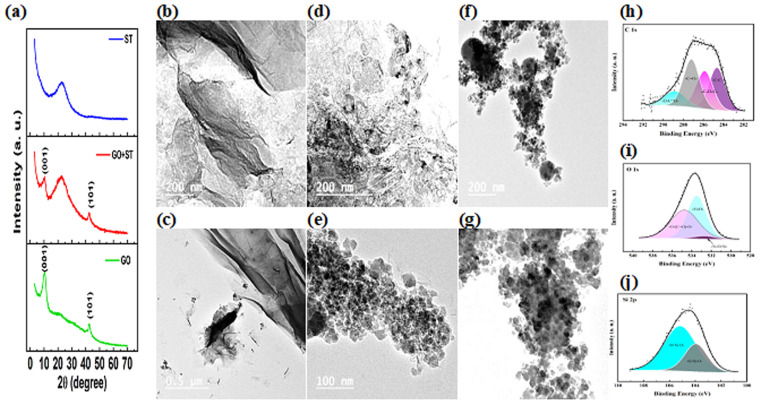
(a) XRD pattern of ST, rGO and rGO-ST, HR-TEM images of the GO (b and c), silica template (d and e), and rGO-ST (f and g), high-resolution XPS spectra of the C 1s (h), O 1s (i), and Si 2p (j) on the surface of the mixed support rGO-ST.

The X-ray powder diffraction patterns of the ST, graphene, and graphene oxide combined with the ST are represented in Fig. 1(a). Regarding the XRD pattern of ST, it showed a single broad reflection at 2*θ* around 22°, and that is equivalent to the average pore–pore correlation distance in the small-angle region, which is consistent with the mesoporous structures of the silica spheres.^39^ The XRD pattern of graphene oxide reflected two peaks at 2*θ* equal to 10.41° and 42.49°. The sharp peak at 10.41° representing the (001) GO layers the oxidation of graphite is confirmed by this diffraction peak that indicated the presence of oxygen-rich functional groups.^40,41^ The 42.49° diffraction peak corresponds to the (101) crystallographic planes of graphitic materials.^42^ The XRD pattern of the mixed support (GO and ST) reflected three diffraction peaks at 2*θ* equal to 10.09°, 22.57°, and 42.65° and this confirmed the presence of both supports, it could be noticed that the intensity of (001) GO peak is decreased because the presence of ST that covered the surface of GO (this will be further confirmed by TEM analysis).

The morphology and particle distribution of different prepared supports and electrocatalysts are determined using TEM analysis. Fig. 1(b–g) represents the TEM images of the GO (b and c), silica template (d and e), and a mixture of both (f and g) under different magnifications. GO showed a tulle-like structure, typically smooth and wrinkled across its surface owing to its oxygenated functional groups.^43^ It can also be seen that the mono- or multi-layered GO particles are created using thin semi-transparent sheets.^44^ Furthermore, TEM images of GO have transparent sheet structures.^42^ It could be noticed that obtained GO is composed of a smooth planar structure with thin individual layers. However, the areas in GO images that have lower brightness may be attributed to the elastic corrugations.^45^

Fig. 1(d and e) represents TEM images of silica samples, revealing that the obtained silica particles are mesoporous; however, neither uniform morphology nor is ordered mesostructure achieved. TEM images confirmed the formation of partially-bridged hollow nanospheres.^46^ Moreover, the SiO_2_ material is composed of round-shape particles on their surface with smaller-sized particles.^47^ The mixed support of GO and silica templates TEM images are displayed in Fig. 1(f and g). It is noticed that the presence of apertures and slits on the GO surface is an indication of the existence of silica. A higher magnification image of the mixed support showed the dispersal of mesoporous silica on the outer surfaces of the GO (Fig. 1g) indicating good dispersion of silica particles on the graphene oxide surface.

**Fig. 2:**
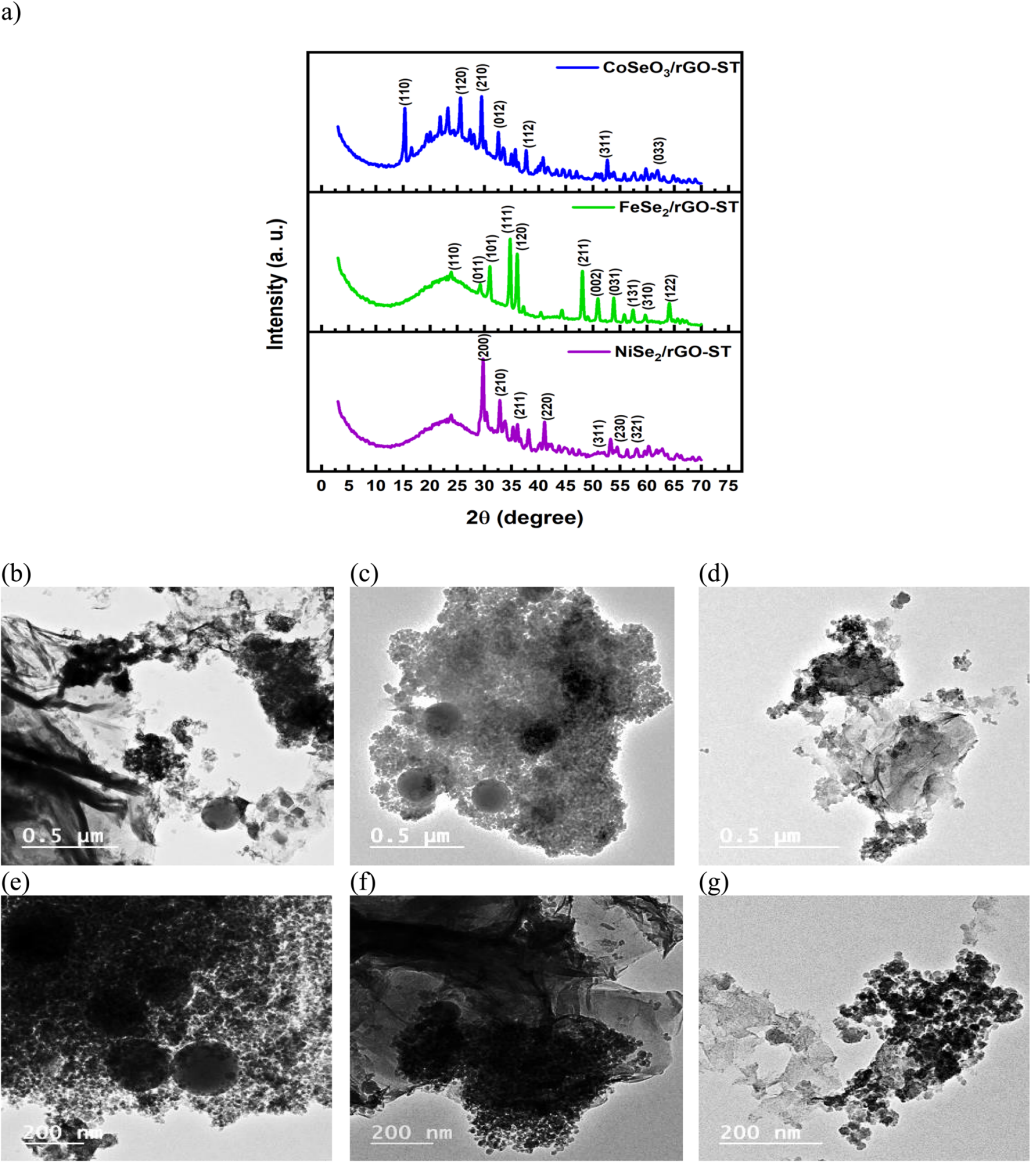
XRD analysis of the electrocatalyst (a), HR-TEM analysis of NiSe_2_/rGO-ST (b and c), CoSeO_3_/rGO-ST (d and e), and FeS_2_/rGO-ST (f and g).

The XPS analyses of the mixed support rGO-ST are shown in Fig. 2(h–j). The C 1s XPS spectra (292.18–281.88 eV) could be deconvoluted into four peaks that attributed to the C–C, C–O–C, O–C=O, and C=O at binding energies of 284.63, 285.86, 288.88 and 287.18 eV, respectively as shown in Fig. 2(h). The O 1s is also deconvoluted into three peaks at 533.48, 532.95, and 534.74 eV that are assigned to Si–OH, Si–O–Si, and O–(C=O)–O, respectively, as indicated in Fig. 2(i).^48^ To further investigate the bonding mode of Si in the support material, the high-resolution Si 2p (109.08–100.68 eV) spectrum is deconvoluted into two peaks at 103.94 and 105.17 eV as illustrated in Fig. 2(j), they are ascribed to SiO_2_. These results imply the doping of ST in the rGO.^49^

#### 3.1.2. Physical characterization of electrocatalyst

Fig. 2(a) illustrated the XRD patterns of prepared selenium-transition metals supported on mixed rGO and ST, it could be noticed that there is not any obvious peak of GO, rGO, or ST in the XRD patterns. We could say that after hydrothermal reaction GO is reduced to rGO or this may be due to the presence of transition metal particles on the surface of the support material that indicates the complete coverage of the surface with the electrocatalysts particles. Furthermore, there are no diffraction peaks of any impurities in all the XRD curves which reflected the formation of a pure phase of prepared electrocatalysts. The diffraction peaks of the FeSe_2_ XRD diffraction curve are completely consistent with the orthorhombic FeSe_2_. Peaks at around 23.83°, 29.25°, 31.07°, 34.75°, 36.07°, 48.08°, 49.00°, 53.82, 57.37°, 59.65° and 64.02° can be marked as diffraction peaks of the orthorhombic FeSe_2_ (110), (011), (101), (111), (120), (211), (002), (031), (131), (310) and (122), respectively.^50^ NiSe_2_ electrocatalysts displayed the diffraction peaks at 2*θ* of 29.86°, 33.79°, 36.79°, 42.43°, 50.56°, 55.30°, and 57.25°, those peaks are standard for the (200), (210), (211), (220), (311), (230) and (321) crystal planes.^51^ Regarding the diffraction curve of CoSeO_3_, it had main characteristic peaks at 15.38°. 25.62°, 29.53°, 32.64°, 37.67°, and 52.63° are all observed and matched well with the crystal planes of (110), (120), (210), (012), (112), (311), and (033).^52^

TEM analysis of NiSe_2_, CoSeO_3,_ and FeS_2_ supported on rGO-ST are performed, and their results are presented in Fig. 2(b–g) respectively, all the nanoparticles are quite fine (around 20 nm in size) where each nanocrystal is connected tightly with its neighboring,^53^ the nanocrystals are composed of irregularly shaped nanoparticles that distributed homogenously on the rGO-ST support. It is also noticed that the ST particles are shown in the form of large dark gray circles furthermore, the wrinkles of reduced graphene could also be observed in all samples indicating well hybridization with MSe_*x*_ nanoparticles.^54^ Heavy distribution of nanoparticles on the mixed support is observed for NiSe_2_/rGO-ST and CoSeO_3_/rGO-ST while it is not the case in FeS_2_/rGO-ST images where there are larger areas of the support without the electrocatalysts nanoparticles. It is well known that nano-scale size and the coverage of the nanoparticles on the support in a dense way provide multiple catalytic sites.

**Fig. 3:**
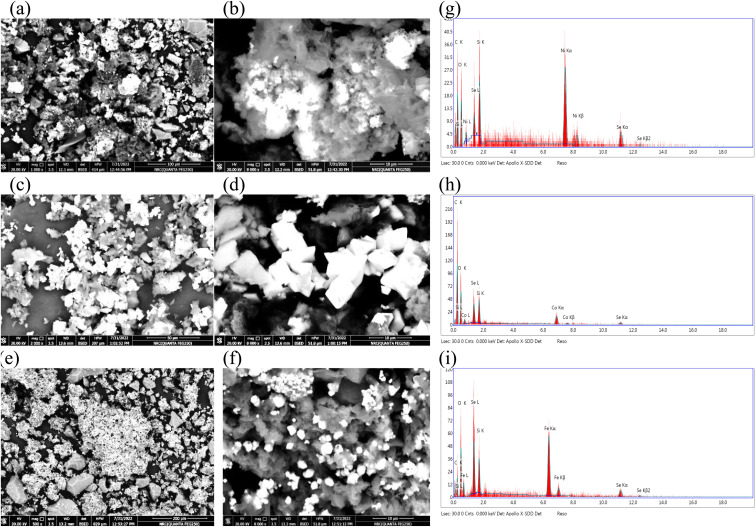
SEM & EDAX analysis of NiSe_2_/rGO-ST (a, b and g), CoSeO_3_/rGO-ST (c, d and h), and FeSe_2_/rGO-ST (e, f and i).

Fig. 3(a–f) represents the SEM images of different electrocatalysts, by reference to the morphology of prepared electrocatalysts, lots of nanoparticles could be easily observed on the substrate, and those particles are stacked together. These nanoparticles are in the form of nanospheres for NiSe_2_/rGO-ST (a and b). Min Zhu *et al.*^55^ synthesized Fe-doped NiSe_2_ nanospheres supported on reduced graphene oxide through low-temperature reflux and solvo thermal process. They found that the presence of reduced graphene as support limits the excess growth of the nanosphere of the electrocatalysts during the synthesis procedure. Moreover, the nanospheres of NiSe_2_ tend to agglomerate in the absence of reduced graphene. In the case of CoSeO_3_/rGO-ST (c and d) and FeSe_2_/rGO-ST (e and f) the particles tended to be cubes this is in agreement with Kaviyarasu^56^ who prepared cubic ZnSe and Balamuralitharan *et al.*^57^ who prepared MnSe_2_ cubes. Abundant mesoporous structures are spreading all over the matrix, which increases the surface area (in agreement with TEM analysis).

Fig. 3(g–i) presents EDAX spectra and elemental composition of MSe/rGO-ST which exhibits peaks for selenium (Se), carbon(C), oxygen (O), silicon (Si), and M where M is Ni, Mn, Co, Zn, and Fe, respectively. These peaks suggest the formation of MSe/rGO-ST. The intensity of different peaks is different. The elemental analysis indicates that selenium and carbon peaks correspond to the substrate while oxygen peaks are from the oxygen present in the atmosphere.^58^ It is also noticed that Ni has intense peaks; this is in coincidence with its corresponding elemental composition.

**Fig. 4:**
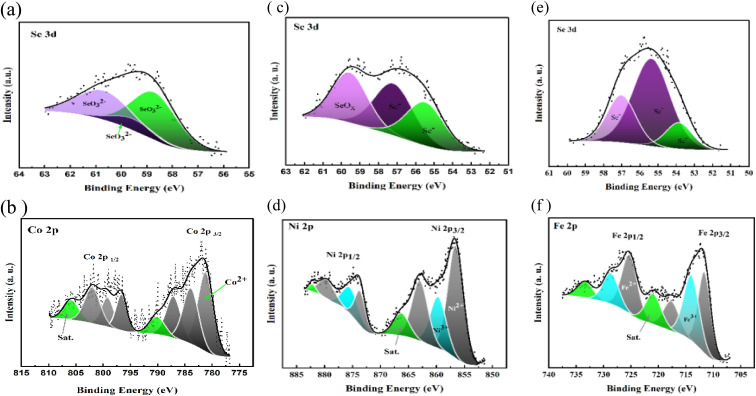
XPS spectra of CoSeO_3_/rGO-ST (a and b), NiSe_2_/rGO-ST (c and d), and FeS_2_/rGO-ST (e and f) electrocatalysts.

The stoichiometry and valences state of each electrocatalyst are further carried out using XPS analyses from the wide survey's spectra of CoSeO_3_/rGO-ST, FeSe_2_/rGO-ST, and NiSe_2_/rGO-ST films that measured over a binding energy range of 0 to 1400 eV and the graphs are displayed in Fig. S1(a–c).† It could be observed that the presence of main photoemission intensity peaks appeared at ∼285.95, 533.63, 104.78, 57.02, 783.13, 713.41 and 858.21 eV. The previous binding energies correspond to the C 1s, O 1s, Si 2p, Se 3d, Co2p, Fe2p, and Ni2p regions, respectively, as the main species in the as-prepared electrocatalysts. The high-resolution XPS spectra of Se 3d spectra regions for all prepared electrocatalysts are presented in Fig. 4(a–c). As displayed in Fig. 4(a) in the case of CoSeO_3_/rGO-ST, exhibited the presence of three fitted peaks at 58.7, 59.51, and 60.67 eV that is in accord with Se–O bonding structures, which indicates the presence of SeO_3_^2−^ as previously illustrated.^59,60^ Whereas, Fig. 4(b and c) showed that there are monovalent (Se^−^) and divalent (Se^2−^) oxidation states of selenium, in addition to the existence of SeO_*x*_ (58.29 eV) that is caused by surface oxidation.^61^ As shown in FeSe_2_/rGO-ST, the characteristic oxidation state of Se^−^ is assigned to binding energies of ∼54.53 and ∼56.6 eV.^62,63^ Moreover, the atomic Se^−2^ corresponds to ∼53.84 eV and 57.37 eV in the case of NiSe/rGO-ST.^56^ The high-resolution XPS spectra of Co2p, Fe 2p, and Ni 2p are also separately presented in Fig. 4(d and e), respectively. The Co 2p spectrum as illustrated in Fig. 2(t) is deconvoluted into two spin–orbit doublets (Co 2p_3/2_ at 776.78 eV and Co 2p_1/2_ at 795.8 eV) along with two respective satellite peaks, as reported previously.^64,65^ Besides, the occurrence of six major peaks positioned at 781.2, 798.48, 796.3, 783.91, 787.09, and 801.98 eV are correlated to Co^2+^ ions.^66^ Furthermore, the prominent satellite peaks are located at 790.13 and 805.84 eV which are attributed to the coexistence of Co^2+^.^5,67^ Similarly, the Fe 2p spectrum reveals two spin–orbit doublets for Fe 2p_3/2_ and Fe 2p_1/2_ at 706.98 and 737.48 eV, respectively. The first doublet is deconvoluted at 711.62, 713.99 and 720.93 eV, indicating the presence of Fe^2+^.^68^ The second one is also deconvoluted at 717.65 and 728.67 eV which can be assigned to Fe^3+^.^69^ Moreover, the presence of two broad satellite peaks at 725.27 and 733.36 eV (Fig. 2(u)). In the case of the Ni 2p_3/2_ and Ni 2p_1/2_ spectra are correlated to the binding energies of 856.58 and 873 eV, respectively, as indicated in Fig. 4(f). In addition, the existence of shake-up satellite peaks at binding energies of 866.28 and 882.15 eV.^70,71^ Furthermore, the obtained peaks at 856.58, 859.72, and 874.99 eV are accredited to Ni^2+^.^72,73^ Whereas, 862.96 and 873.23 eV are assigned to Ni^3+^.^74–76^ Ni^3+^ may be produced by the low coordination or partial oxidation of Ni ions at the surface.^77^ Consequently, the Ni selenide mainly show a divalent state.

Moreover, for all specimens, to further probe the bonding mode of Si as illustrated in Fig. S2(a),† the fitted high-resolution Si 2p spectrum region is deconvoluted into two distinguishable peaks at ∼103.98 and ∼105.05 eV that ascribed to –C–Si–O bond and O–Si–O bonds of SiO_2_, respectively.^49^ Significantly, the high-resolution C 1s spectra revealed a major peak fitted at 284.68 eV that corresponded to the C–C/C=C bonds of sp^2^ hybridized graphite carbon atoms in Fig. S2(b).† Another oxygen-containing group that could be assigned to C–O, C–O–C, C=O, O–C=O chemical bonds at 286.08, 286.4, 287.64, 289.42 eV, respectively, these values are consistent with those for rGO.^78–80^ The accompanying O 1s spectra manifest the coexistence of three peaks with binding energies at 530.86, 532.97, and 534.4 eV that could be attributed to the C=O, Si–O–Si, and O–(C=O)–O, respectively, that assists as a nucleus structure on the surface of rGO in Fig. S2(c).† It could be concluded that the XPS results are in line with XRD data.

### 3.2. HER performance

**Fig. 5:**
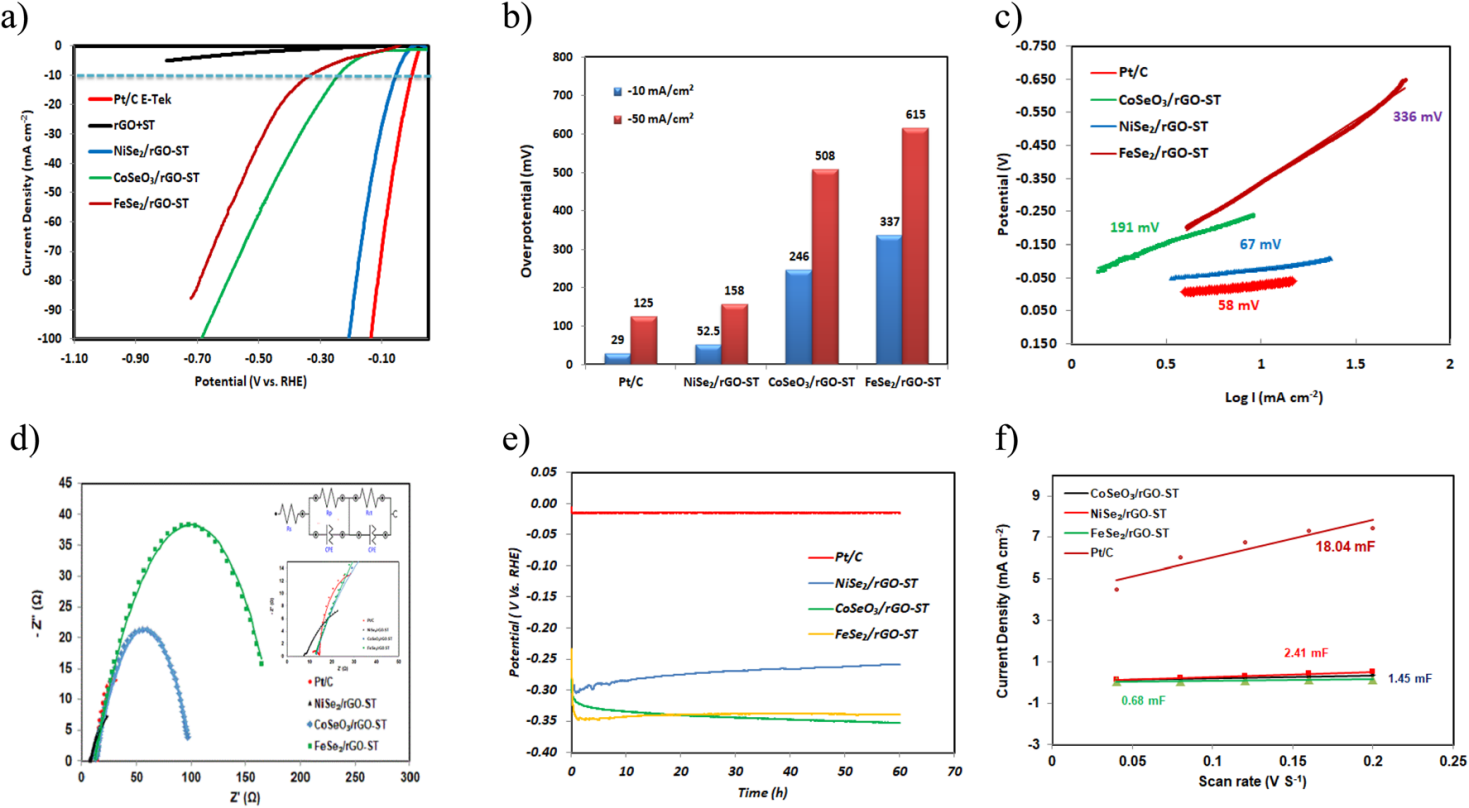
OER performance of the electrocatalysts Pt/C E-TEK, NiSe_2_/rGO-ST, CoSeO_3_/rGO-ST and FeSe_2_/rGO-ST. (a) LSV curves, (b) the comparison of the overpotential, (c) Tafel plots derived from LSV curves, (d) Nyquist plots, (e) chronopotentiometry (CP) curve, and (f) the double-layer capacitance (*C*_dl_) plots.

HER performance of electrocatalysts NiSe_2_/rGO-ST, FeSe_2_/rGO-ST, and CoSeO_3_/rGO-ST is tested in a three-electrode system using 1 M KOH as electrolyte. For comparison, the performance of the mixed support rGO-ST and Pt/C E-TEK is also evaluated under the same conditions. The *iR*-corrected linear polarization curves (LSV) are represented in Fig. 5(a). At a current density of 10 mA cm^−2^, NiSe_2_/rGO-ST had an overpotential of 52 mV, which is somewhat comparable to Pt/C E-TEK (29 mV) and much smaller than those of FeSe_2_/rGO-ST (337 mV), and CoSeO_3_/rGO-ST (246 mV). The linear polarization curve (LSV) without *iR* correction for the electrocatalysts for HER is shown in Fig. S3(a).† Furthermore, *η* values at 50 mA cm^−2^ are 125, 158, 518 and 615 mV for Pt/C, NiSe_2_/rGO-ST, CoSeO_3_/rGO-ST, and FeSe_2_/rGO-ST, respectively as shown in Fig. 5(b). The ESI† (rGO-ST) exhibited poor performance indicating that the improved catalytic activity is related to the metal electrocatalysts.^81^ From the thermodynamics point of view, to have the same current density at lower overpotential means better performance of the electrocatalyst because of less electrical energy consumption. In addition, *η* value at 10 mA cm^−2^ of the NiSe_2_/rGO-ST surpasses that of some recently reported HER catalysts in alkaline medium, for example, nickel diselenide (NiSe_2_) anchored on carbon fiber paper (CFP) (145 mV),^82^ iron-doping NiSe_2_ nano wrinkles (145 mV),^83^ copper-incorporated heterostructures of amorphous NiSe_*x*_/crystalline NiSe_2_ (156.9 mV),^84^ CNTs encapsulating P-doped NiSe_2_ nanoparticles on carbon framework (95 mV),^85^ hybrid nanosheet arrays NiSe_2_/CC-180 (133 mV),^86^ NiO–NiSe_2_ nanosheet-based heterostructures shelled titanium nitride array (115 mV),^87^ NiSe_2_/Ni_2_P@FeP interface nanosheets (113 mV).^88^ To the best of our knowledge, CoSeO_3_/rGO-ST and FeSe_2_/rGO-ST catalyzing HER have been rarely reported in the literature. However, they both have a reasonable and comparable overpotential at 10 mA cm^−2^, for example, Xueying Li *et al.*^89^ prepared different HER electrocatalysts composed of Fe–Co–S nanoflakes grew on the carbon cloth by hydrothermal method and annealing treatment, they found that at the current densities of 10 mA cm^−2^, the overpotential of Co–S/CC, Fe–Co–S/CC-37.5, Fe–Co–S/CC-75, and Fe–Co–S/CC-150 is 330, 320, 260, and 320 mV respectively. Moreover, the Co–BTC (1,3,5-benzene tricarboxylic acid) electrocatalyst prepared by pulsed laser ablation in dimethyl formamide^90^ showed an overpotential of 437 mV toward HER at a current density of 10 mA cm^−2^ in 1.0 M KOH. More performance comparison with recently published non-noble metal-based for HER catalysts is listed in Table 1.

**Table 1:** A performance comparison with recently published non-noble metal-based for HER, OER and water splitting catalysts

Electrocatalyst	HER		OER		Water electrolysis
	Overpotential *η* (mV)@10 mA cm^−2^	Tafel slopes (mV dec^−1^)	Overpotential *η* (mV)	Tafel slopes (mV dec^−1^)	Potential (V)@10 mA cm^−2^
NiSe_2_/rGO-ST (this study)	52.5	67	280@20 mA cm^−2^	236	NiSe_2_-rGO-ST/2NF||FeSe_2_-rGO-ST/NF (1.75)
			400@50 mA cm^−2^		
CoSeO_3_/rGO-ST (this study)	246	191	284@20 mA cm^−2^	127	
			475@50 mA cm^−2^		
FeSe_2_/rGO-ST (this study)	347	363	229@20 mA cm^−2^	106	
			297@50 mA cm^−2^		
NiSe_2_/CF (NSN/CFP)^25^	145	72	280@10mA cm^−2^	81	1.66 (bifunctional)
P-NiSe_2_@N-CNTs/NC^27^	95	82	306@10 mA cm^−2^	61	1.61 (bifunctional)
Se–NiSe_2_/CC^86^	133	128	350@100 mA cm^−2^	190	1.57 (bifunctional)
TiN@NiO–NiSe_2_/CC^87^	115	59 ± 4	240@10 mA cm^−2^	29 ± 3	1.57 (bifunctional)
NiSe_2_/Ni_2_P@FeP^109^	113	73.1	202@10 mA cm^−2^	42.1	1.554 (bifunctional)
Fe–NiSe_2_/CC^110^	—	—	257@10 mA cm^−2^	43	—
NiSe_2_/Ni_2_O_3_/FeOOH^111^	—	—	270@100 mA cm^−2^	54.7	—
10%P–NiSe_2_/CoSe_2_ (ref. 88)	—	—	287@50 mA cm^−2^	68	—
NiSe_2_/FeSe_2_/NiFe (NFS/NFF)^23^	—	—	274@40 mA cm^−2^	57.07	—
Ni_0.7_Fe_0.3_Se_2_/rGO-30%	—	—	265@10 mA cm^−2^	57	—
Ni_0.7_Fe_0.3_Se_2_			325@10 mA cm^−2^	70	
NiSe_2_ (ref. 55)			422@10 mA cm^−2^	79	
Cu-(a-NiSe_*x*_/c-NiSe_2_)/TiO_2_ (ref. 85)	156.9	51.2	339@10 mA cm^−2^	54.2	1.62 (bifunctional)
NiSe_2_/Ni–Fe^26^	145	—	135@10 mA cm^−2^	—	1.58 (bifunctional)
KMF_3_ (M = Co/Fe)^112^	—	—	254@10 mA cm^−2^	37.5	—
Fe_2_OF_4_/nickel foam^113^	—	—	238@10 mA cm^−2^	48	—
Fe–NiCoP^114^	—	—	266@10 mA cm^−2^	75.2	Fe–NiCoP||Pt/C
					1.72
FeSe_2_	—	—	338@10 mA cm^−2^	139	—
CoSe_2_			346@10 mA cm^−2^	65	
NiSe_2_			305@10 mA cm^−2^	91	
NiSe_2_/FeSe_2_ (ref. 115)			256@10 mA cm^−2^	50	
FeSe_2_–CoSe_2_/CoSe_2_ (ref. 116)	—	—	260@10 mA cm^−2^	51.3	—
CoSe_2_@NSC	—	—	364@10 mA cm^−2^	114.78	FeSe_2_/CoSe_2_@NSC||Pt/C
FeSe_2_/CoSe_2_@SC			407@10 mA cm^−2^	110.50	1.57
FeSe_2_/CoSe_2_@NSC^117^			278@10 mA cm^−2^	53.08	
Fe–Co–S/CC^89^	320	82.60	258@10 mA cm^−2^	61.20	1.51 (bifunctional)

As shown in Fig. 5(c) the outstanding HER catalytic activity of NiSe_2_/rGO-ST is further verified by the corresponding Tafel slope of 67 mV dec^−1^ revealing a typical Volmer–Heyrovsky pathway with the Volmer step being the rate-limiting step,^91^ Tafel slopes of FeSe_2_/rGO-ST is 363 mV dec^−1^ and that of CoSeO_3_/rGO-ST is 191 mV dec^−1^. The small Tafel slope of NiSe_2_-rGO-ST will lead to a faster incrimination of the HER rate with increasing overpotential.^92^

The Nyquist plots are represented in Fig. 5(d), and equivalent circuits of the electrocatalysts are shown in the inset of Fig. 5(d). The *R*_s_ (resistance of the solution), *R*_ct_ (resistance of the charge transfer), and CPE (constant phase element) values are represented in Table S1.† The charge transfer resistance values (*R*_ct_) are determined by the semicircular diameter in the high-frequency region.^93,94^ The *R*_ct_ values for Pt/C E-TEK, NiSe_2_/rGO-ST, CoSeO_3_/rGO-ST, and FeSe_2_/rGO-ST and are 27.29, 40.07 and 87.41, 168.90 Ω respectively in 1 M KOH. From a general point of view, small values of *R*_ct_ mean good contact between nano electrocatalysts and supporting materials. Moreover, NiSe_2_/rGO-ST has the smallest *R*_ct_ value, in other words, it has the fastest charge transfer ability among tested electrocatalysts.^93,94^

Fig. 5(e) shows the chronopotentiometry curve of different electrocatalysts at an overpotential of −10 mV in 1 M KOH solution for 3600 s. The current density showed only a little deterioration, which might be attributed to H^+^ consumption or residual H_2_ bubbles on the electrode surface, which hampered the process.^81^ The NiSe_2_/rGO-ST showed the largest and the most stable current when compared to CoSeO_3_/rGO-ST and FeSe_2_/rGO-ST. As a result, the NiSe_2_/rGO-ST showed superior stability for the HER in the alkaline electrolyte.

To gain further insight into HER activity, we determine the electrochemical sensitive area (ECSA) which is directly affected by the number of active sites for the HER.^95^ The ECSA is calculated by measurement of the capacitance as it is linearly proportional to the double-layer capacitance (*C*_dl_).^96^ ECSA is calculated according to the following equation:^97^

1 ECSA (cm^2^) = capacitance (μF)/40 μF cm^−2^

The electrochemical active surface area is estimated using the simple cyclic voltammetry (CV) method. Fig. S4(a–c)† represented CVs of the prepared electrocatalysts in the potential region from (0.67 to 0.77 V *vs.* RHE) at different scan rates of 40–200 mV s^−1^. The double-layer capacitances (*C*_dl_) for each electrocatalyst are directly proportional to the surface area. It is determined by plotting Δ*J* = *j*_a_ − *j*_c_ at a given potential (0.72 V *vs.* RHE) against the CV scan rates and illustrated in Fig. S4† ^98^ in which the slope is equal to twice that of *C*_dl_.^99^ A linear plot with slope values of 18.04, 2.41, 1.45, and 0.68 mF cm^−2^ for Pt/C E-TEK, NiSe_2_/rGO-ST, CoSeO_3_/rGO-ST, and FeSe_2_/rGO-ST, respectively as shown in Fig. 5(f). These *C*_dl_ values resemble those obtained by Zhu *et al.*^100^ who obtained a *C*_dl_ value of 2.77 mF cm^−2^ for CoSeO_3_/rGO-ST and 1.93 mF cm^−2^ for NiSe_2_/rGO-ST.

Pt-E-TEK retained a high ECSA of 270.75 cm^2^ while those of NiSe_2_/rGO-ST, CoSeO_3_/rGO-ST and FeSe_2_/rGO-ST are 36.12, 14.25 and 12.32 cm^2^. The superior HER catalytic performance of NiSe_2_/rGO-ST could be due to the large electrochemically active surface area and subjected active sites.

Based on the above findings, we may conclude that NiSe_2_/rGO-ST is a potential low-cost catalyst for the hydrogen evolution process in an alkaline medium depending on its remarkable overpotential, Tafel slope, electrochemical surface area, and stability values.

### 3.3. OER performance

**Fig. 6:**
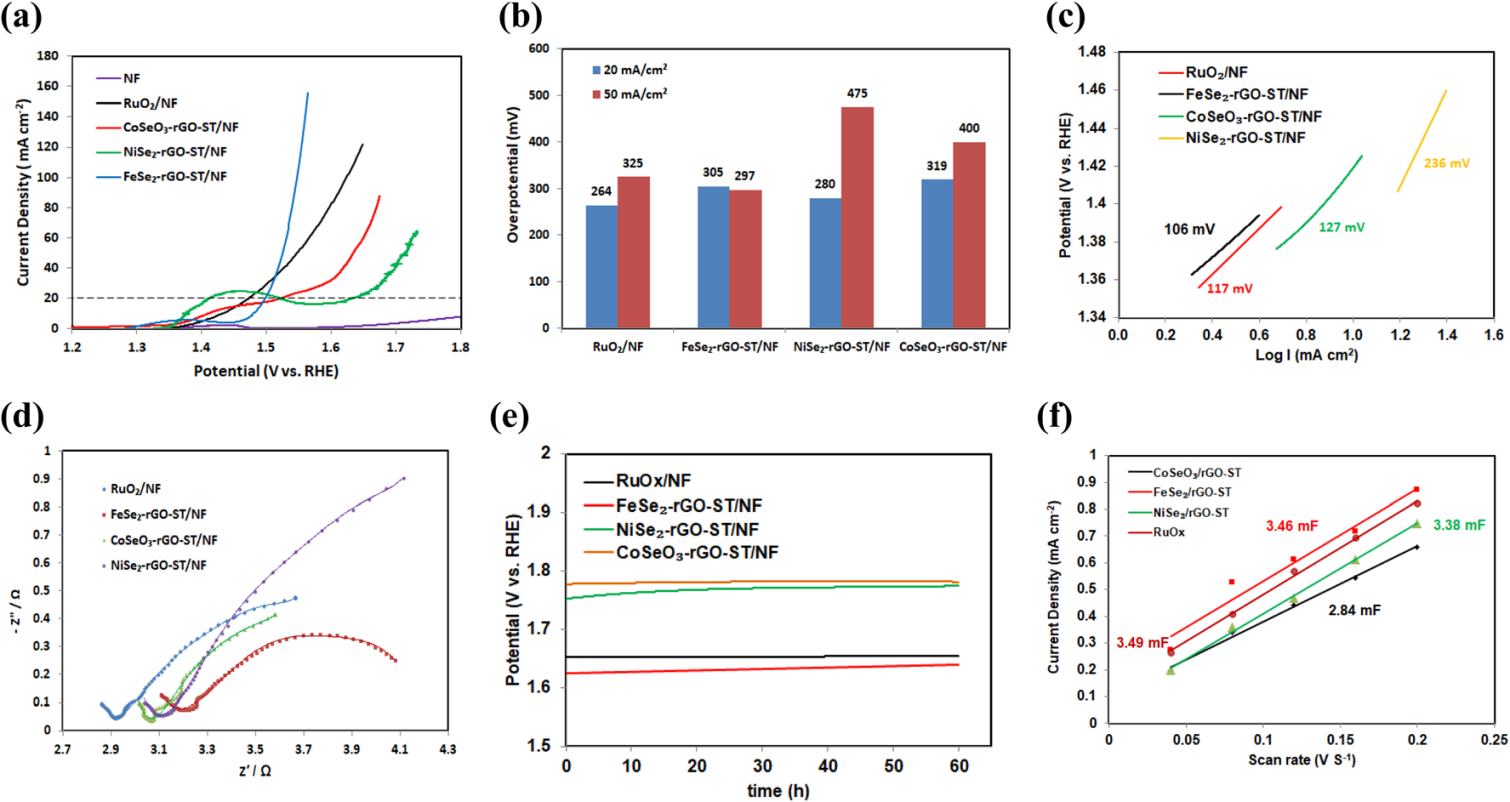
OER performance of the electrocatalysts RuO_*x*_, NiSe_2_/rGO-ST, CoSeO_3_/rGO-ST, and FeSe_2_/rGO-ST. (a) LSV curves, (b) the comparison of the overpotential, (c) Tafel plots derived from LSV curves, (d) Nyquist plots, and (e) chronopotentiometry (CP) curve at 30 mA cm^−2^.

OER activities of prepared electrocatalysts are further investigated using linear sweep voltammetry (LSV). Similar measurements are also taken for bare NF and RuO_*x*_/NF with the same loading. Their linear sweep voltammetry (LSV) curves *vs.* the reversible hydrogen electrode (RHE) scale is shown in Fig. 4(a). All potentials shown are corrected for the ohmic potential drop, and current densities are calculated using the projected geometric area of the electrode. As shown in Fig. 6(a), NiSe_2_-rGO-ST/NF exhibits an oxidation peak around 1.35 V *vs.* RHE before H_2_O oxidation, which is attributed to the transformation of Ni^II^ to Ni^III^ species.^101,102^ The linear polarization curve (LSV) without *iR* correction for the electrocatalysts for OER is shown in Fig. S3(b).† The overpotential is affected by metal insertion in the MSe_*x*_-rGO-ST/NF electrocatalyst. FeSe_2_-rGO/NF demonstrated significantly better OER performance than RuO_*x*_/NF, NiSe_2_-rGO-ST/NF, and CoSeO_3_-rGO-ST/NF, with overpotential at 20 mA cm^−2^ of 270, 229, 280, and 284, respectively. At 50 mA cm^−2^, the overpotential for RuO_*x*_/NF, FeSe_2_-rGO/NF, NiSe_2_-rGO-ST/NF, and CoSeO_3_-rGO-ST/NF are 325, 297, 400, and 475, respectively as shown in Fig. 6(b).

The electrocatalytic performances of FeSe_2_-rGO-ST/NF for OER in 1 M KOH are compared with previously reported literature. The overpotential value at 10 mA cm^−2^ for different iron composite electrocatalyst *e.g.* FeSe_2_ supported on CoSe using hydrothermal and selenization process is 183 mV,^29^ Fe–Co–S/CC-37.5 and Fe–Co–S/CC-150 using hydrothermal process is 320 mV,^89^ Fe–Mo supported on Te is 300 mV,^103^ Fe(Se_0.5_S_0.5_)_2_ using hydrothermal process is 247 mV,^104^ FeSe_2_ using hydrothermal process is 330 mV,^105^ FeP and FeCoNiP prepared by chemical reduction and phosphorization process are 325 and 200 mV, respectively,^106^ Fe_0.5_Co_0.5_Se_2_ supported on carbon fiber cloth prepared by solvo thermal and selenization process is 290 mV,^107^ NiFeSe_*x*_ supported on carbon fiber cloth prepared by electrodeposition followed by solvo thermal process is 310 mV.^108^ Additional LSV and Tafel slope comparison of recently published non-noble metal-based for OER catalysts are shown in Table 1.

The kinetics of OER is estimated using corresponding Tafel plots (*η versus* log(*j*)) for these electrodes (Fig. 6(c)). The Tafel slope for Fe–Se_2_-rGO-ST/NF is 106 mV dec^−1^, which is less than that of RuO_*x*_/NF (117 mV dec^−1^), CoSeO_3_-rGO-ST/NF (127 mV dec^−1^) and Ni–Se_2_-rGO-ST/NF (236 Ni–Se_2_-rGO-ST), implying a faster OER rate for Fe–Se_2_-rGO-ST/NF electrode.

The Nyquist plots (Fig. 6(d)) show that FeSe_2_-rGO/NF has the smallest charge transfer resistance (*R*_ct_) of 4.31 Ω, which is much lower than those of RuO_*x*_/NF (4.685 Ω), NiSe_2_-rGO-ST/NF (6.627 Ω) and CoSeO_3_-rGO-ST/NF (4.425 Ω), suggesting its high charge transport efficiency of FeSe_2_-rGO/NF in OER process.

In order to evaluate the stability of the electrocatalyst, the OER stability is tested by chronoamperometry (CA). Fig. 6(e) shows that all the electrocatalyst indicates negligible deterioration, and better stability during the process of OER after the stability test of 60 h at the current density of 30 mA cm^−2^.

To determine the precise number of active sites engaged in the electrochemical process, the ESCAs of the electrocatalyst for OER are gathered. The double-layer capacitance (*C*_dl_), which is correlated positively with the ESCAs of the catalysts, can be measured at different scan rates in the potential area between (1.2 and 1.3 V *vs.* RHE) to determine the *C*_dl_ value (Fig. S5†). According to Fig. 6(f), Fe–Se_2_-rGO-ST/NF has a substantially higher *C*_dl_ value (3.46 mF) than other catalysts, which shows that under the same loading situation, Fe–Se_2_-rGO-ST/NF has more active sites.

### 3.4. Water splitting performance

**Fig. 7:**
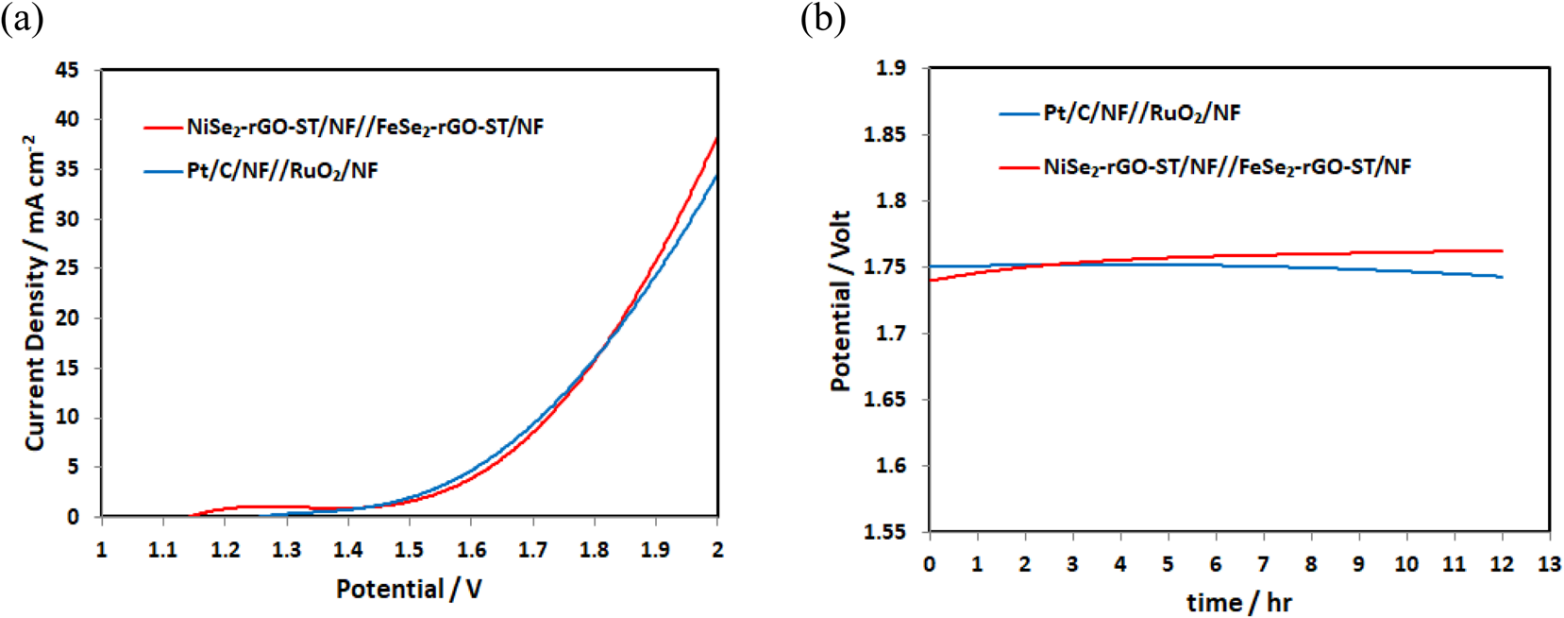
(a) LSV curves of Pt/C/NF||RuO_2_/NF and NiSe_2_-rGO-ST/NF||FeSe_2_-rGO-ST/NF, (b) the chronoamperometry curve of Pt/C/NF||RuO_2_/NF and NiSe_2_-rGO-ST/NF||FeSe_2_-rGO-ST/NF at 10 mA cm^−2^.

Stimulated by the best performance of the NiSe_2_-rGO-ST/NF for HER and FeSe_2_-rGO-ST/NF for OER, the water electrolysis cell is assembled by using FeSe_2_-rGO-ST/NF as anode and NiSe_2_-rGO-ST/NF as cathode, for comparison Pt/C/NF and RuO_2_/NF is tested as cathode and anode, and the overall water splitting performance is further investigated in 1 M KOH. As shown in Fig. 7(a), the as-assembled NiSe_2_-rGO-ST/NF||FeSe_2_-rGO-ST/NF system indicates excellent overall water-splitting performance with low cell voltages of 1.75 V at a current density of 10 mA cm^−2^, which is close to the value of a noble metal-based electrocatalysts Pt/C/NF||RuO_2_/NF. Furthermore, the NiSe_2_-rGO-ST/NF||FeSe_2_-rGO-ST/NF system exhibits outstanding electrocatalytic stability with a negligible potential decline at the constant applied current density of 10 mA cm^−2^ for 12 h as shown in Fig. 7(b). The excellent water decomposition activity and stability of NiSe_2_-rGO-ST/NF||FeSe_2_-rGO-ST/NF system indicate a promising application potential. The overall water splitting performance is compared with updated published data is listed in Table 1.

## 4. Conclusion

In conclusion, we have used a simple one-pot solvo thermal method synthesis strategy to construct a bifunctional electrocatalyst of metal selenium nanoparticles (M = Ni, Co & Fe) anchored on the surface of reduced graphene oxide and silica template (rGO-ST). The combined benefits include the improved mass-transport capacity of pores, high conductivity, and an abundance of numerous active sites of NiSe_2_-rGO-ST and FeSe_2_-rGO-ST enabling to deliver of remarkable bifunctional catalytic activity towards HER (52.5 mV at 10 mA cm^−2^), and OER (235 mV at 10 mA cm^−2^). The assembled electrolyzer with the optimized NiSe_2_-rGO-ST/NF||FeSe_2_-rGO-ST/NF electrodes only needs 1.75 V at 10 mA cm^−2^. The high performance for water splitting is even comparable to those of noble metal catalysts.

## Conflicts of interest

There are no conflicts to declare.

## Supplementary Material

RA-013-D3RA01945D-s001
