# Supplementary material for: Selenium-transition metal supported on a mixture of reduced graphene oxide and silica template for water splitting
Source: RSC Adv. 2023 May 25;13(23):15856–71. doi: 10.1039/d3ra01945d (PMC10209667; doi:10.1039/d3ra01945d)
Supplement: RA-013-D3RA01945D-s001 [file RA-013-D3RA01945D-s001.pdf]

This journal is © The Royal Society of Chemistry 2023

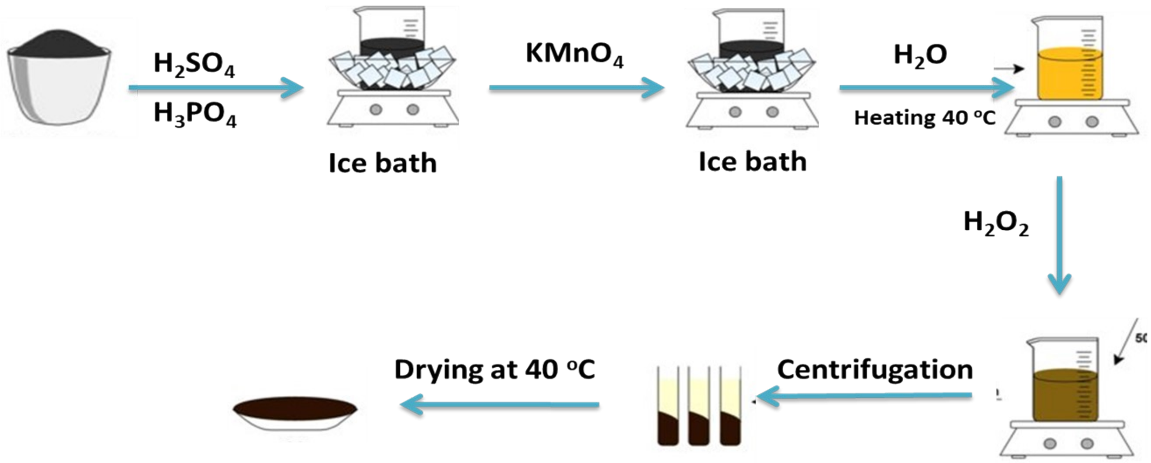

Scheme 1: Procedure of Graphene oxide preparation

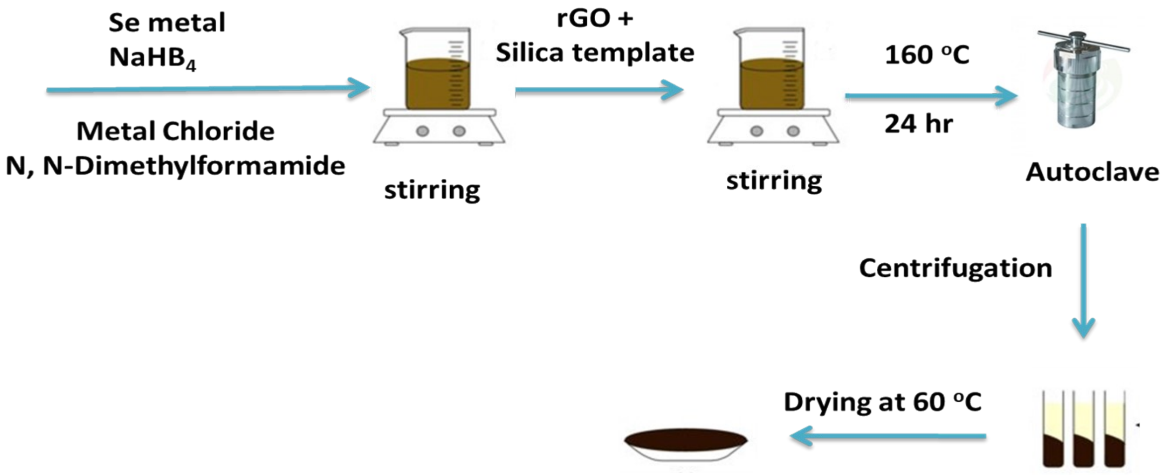

Scheme 2: Procedure of Selenium-transition metal supported on rGO and ST Preparation

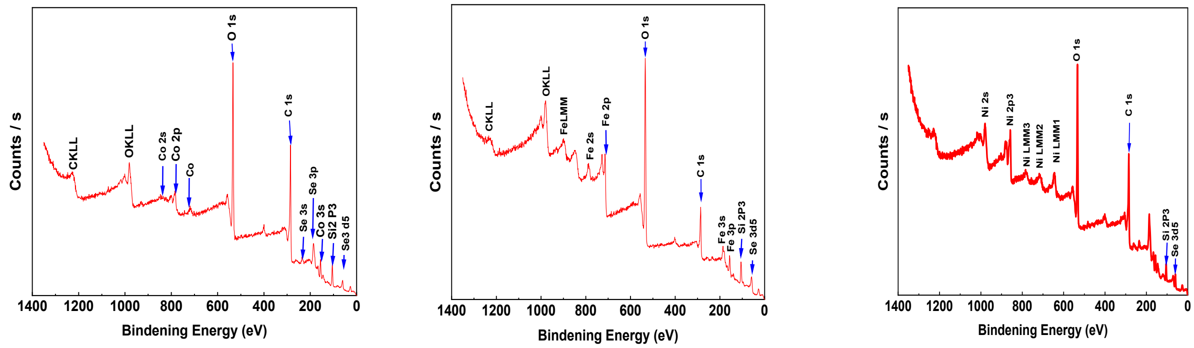

Fig. S1. High-resolution XPS spectra survey of (a) $CoSeO_3$/rGO-ST , (b) $FeSe_2$ /rGO-ST, (c) NiSe/rGO-ST electrocatalysts.

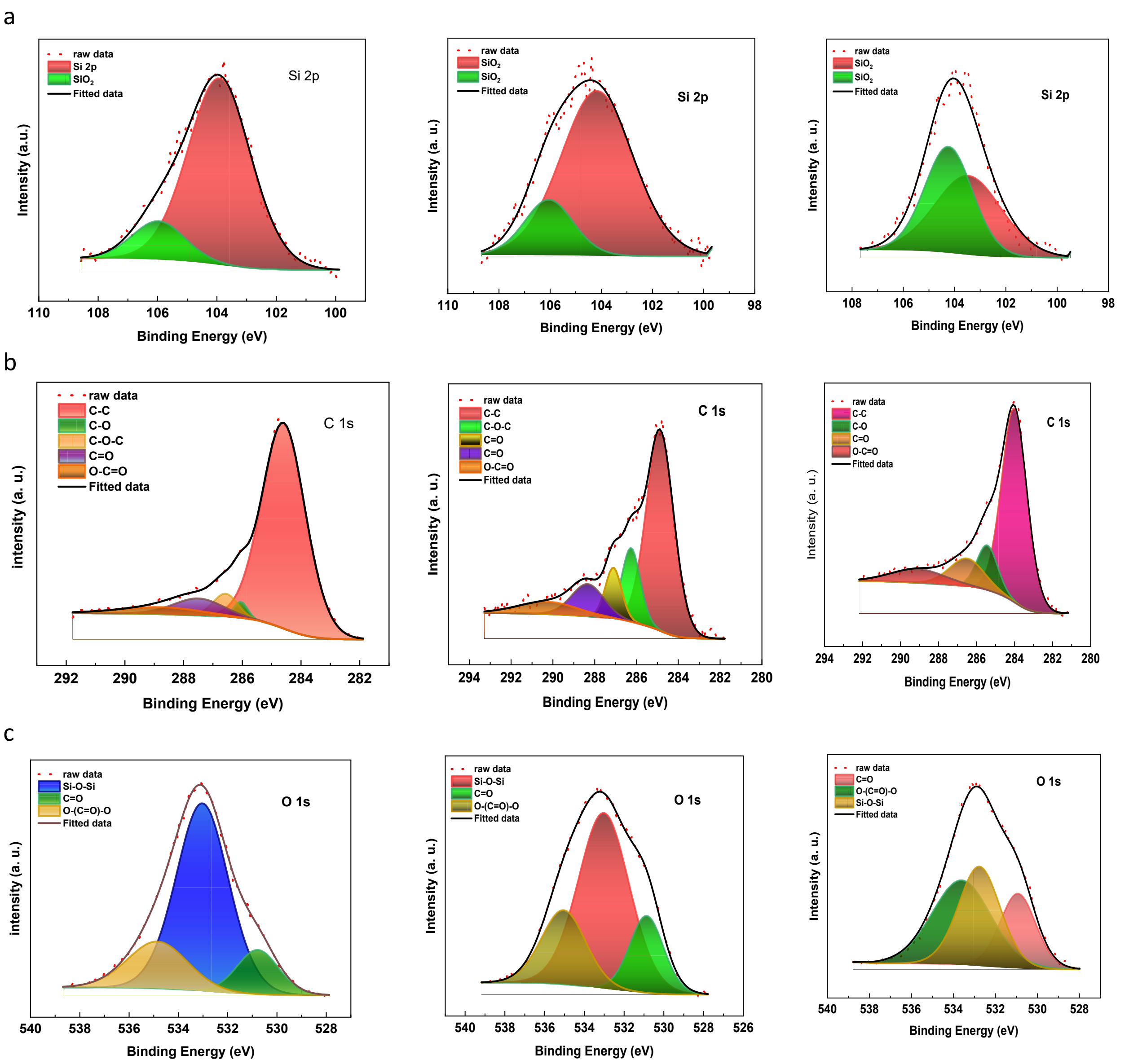

Fig. S2. High-resolution XPS spectra of (a) Si2p , (b) C 1s, (c) O 1s of of $CoSeO_3$/rGO-ST, $FeSe_2$ /rGO-ST and NiSe/rGO-ST electrocatalysts.

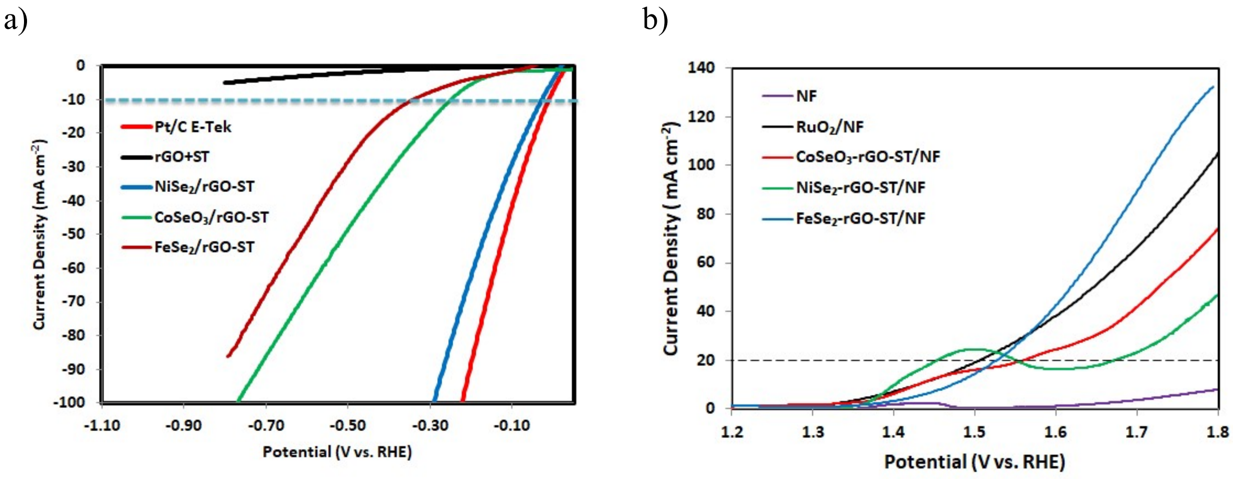

Fig.S3: LSV curve without IR correction of the electrocatalysts (a) HER curves, (b) OER

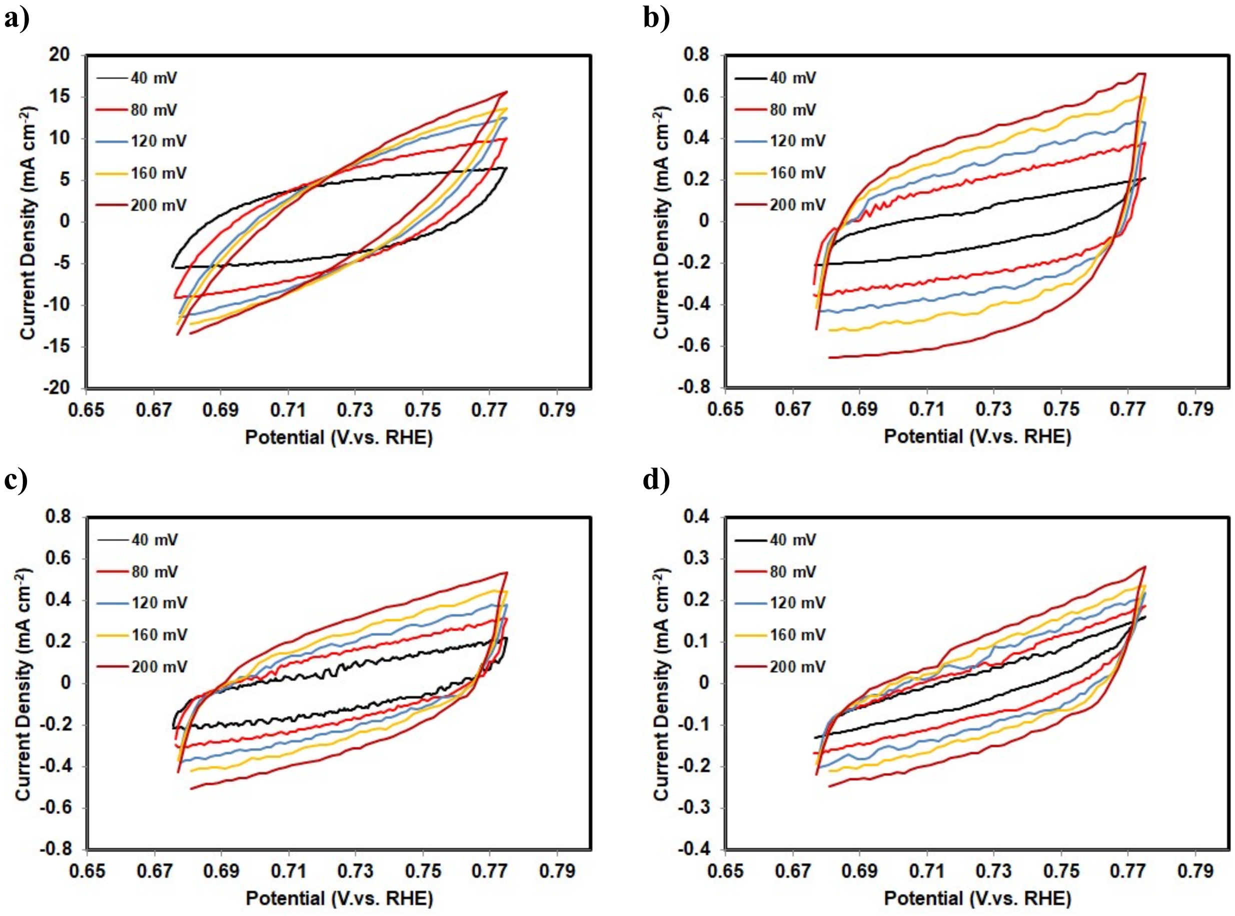

Fig. S4: Non-faradaic CV scans of Pt/C E-TEK a), $NiSe_2$/rGO-ST b), $CoSeO_3$/rGO-ST c) and $FeSe_2$/rGO-ST d) for HER at different scan rate

.a)

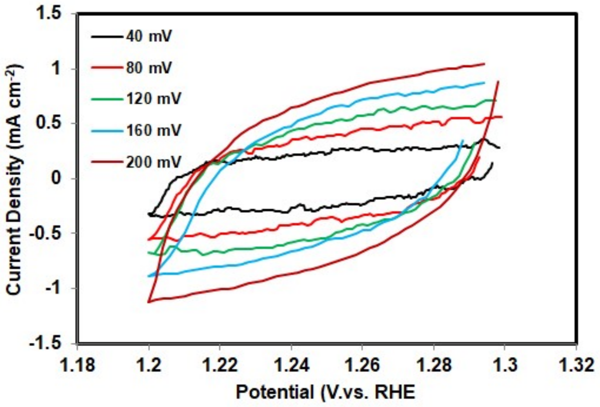

b)

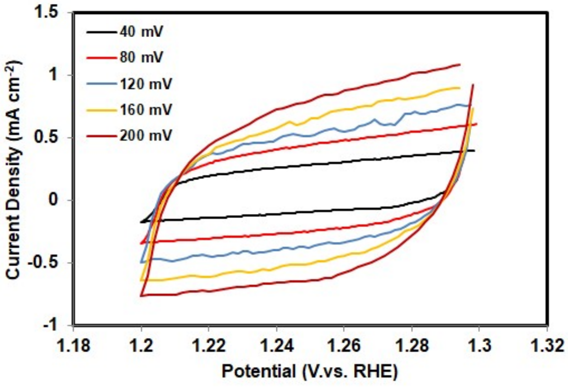

c)

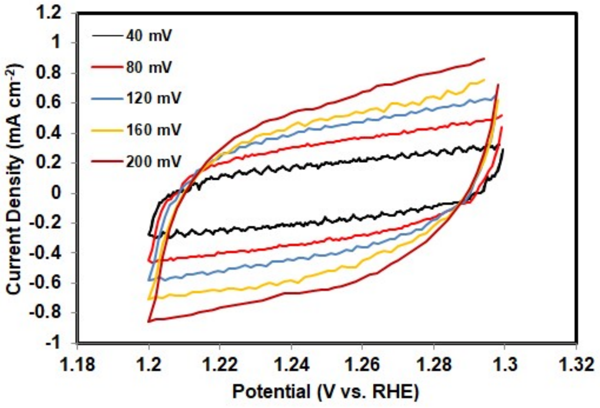

d)

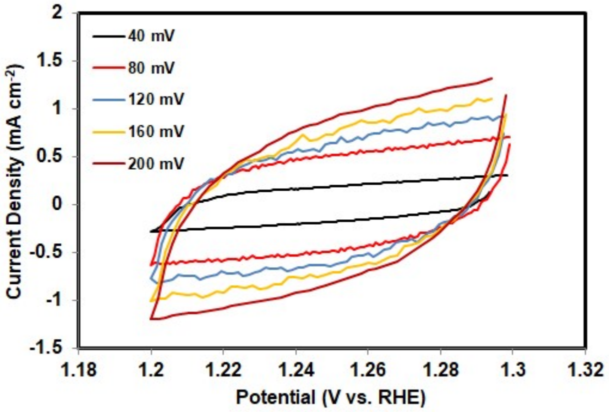

Fig. S5: Non-faradaic CV scans of RuOx a), $NiSe_2$/rGO-ST b), $CoSeO_3$/rGO-ST c) and $FeSe_2$/rGO-ST d) for OER at different scan rate

Table S1: The Rs, CPE, and Rct values for Pt/C E-TEK, $NiSe_2$/rGO-ST, $CoSeO_3$/rGO-ST and $FeSe_2$/rGO-ST

Electrocatalyst	Rs (Ω)	Rp	CPE	Rct	CPE
**Pt/C**	10.52	4.0811	0.00080503	27.296	0.056829
**$NiSe_2$/rGO-ST**	12.844			87.412	0.00092401
**$CoSeO_3$/rGO-ST**	7.5243	0.72285	0.00031961	40.079	0.051609
**$FeSe_2$/rGO-ST**	12.46			168.9	0.0011552
